# Hemispheric Asymmetry of Human Brain Anatomical Network Revealed by Diffusion Tensor Tractography

**DOI:** 10.1155/2015/908917

**Published:** 2015-10-11

**Authors:** Ni Shu, Yaou Liu, Yunyun Duan, Kuncheng Li

**Affiliations:** ^1^State Key Laboratory of Cognitive Neuroscience and Learning and IDG/McGovern Institute for Brain Research, Beijing Normal University, Beijing 100875, China; ^2^Center for Collaboration and Innovation in Brain and Learning Sciences, Beijing Normal University, Beijing 100875, China; ^3^Department of Radiology, Xuanwu Hospital, Capital Medical University, Beijing 100053, China

## Abstract

The topological architecture of the cerebral anatomical network reflects the structural organization of the human brain. Recently, topological measures based on graph theory have provided new approaches for quantifying large-scale anatomical networks. However, few studies have investigated the hemispheric asymmetries of the human brain from the perspective of the network model, and little is known about the asymmetries of the connection patterns of brain regions, which may reflect the functional integration and interaction between different regions. Here, we utilized diffusion tensor imaging to construct binary anatomical networks for 72 right-handed healthy adult subjects. We established the existence of structural connections between any pair of the 90 cortical and subcortical regions using deterministic tractography. To investigate the hemispheric asymmetries of the brain, statistical analyses were performed to reveal the brain regions with significant differences between bilateral topological properties, such as degree of connectivity, characteristic path length, and betweenness centrality. Furthermore, local structural connections were also investigated to examine the local asymmetries of some specific white matter tracts. From the perspective of both the global and local connection patterns, we identified the brain regions with hemispheric asymmetries. Combined with the previous studies, we suggested that the topological asymmetries in the anatomical network may reflect the functional lateralization of the human brain.

## 1. Introduction

The brain exhibits asymmetry in both macroscopic structure and microscopic cytoarchitecture. Moreover, many studies have revealed the anatomical asymmetry corresponding with functional lateralization [1–3]. For example, leftward asymmetries in the size of regions are involved in language and auditory processing, such as planum temporale [4–7], sylvian fissure [8], and Heschl's gyrus [4, 9, 10], consistent with left-hemispheric dominance for language [11]. Recently, functional neuroimaging studies have established hemispheric specificity for a range of language, motor, and spatial tasks [12]. Structural MRI studies have revealed the structural asymmetries in the cortical thickness or gray matter volumes of various brain regions [13, 14]. Using diffusion tensor imaging (DTI) techniques, researchers have also identified the leftward asymmetries of some white matter tracts, such as cingulum bundles [15, 16] and arcuate fasciculus [2, 3, 17–21].

These previous studies examined the structural or functional asymmetries of some specific brain regions or the anatomical connections between them. The asymmetries of the gray matter or white matter were analyzed from the local regional attributes. Recently, network model was proposed as a useful tool for investigating the structural organization and functional mechanisms of the human brain [22–31]. Graph theory approaches the analyses of complex network that could provide a new powerful way of quantifying the brain's structural and functional systems. With the network models, more and more studies have revealed that the structural and functional networks of the human brain exhibit small-world attributes [23–26, 28, 30, 32–34] and modular structure [35–37]. Using diffusion MRI, several studies have proposed different methods to construct the brain anatomical network [25, 32, 33, 36, 38, 39]. All these studies revealed that the cortical networks of the human brain have a “small-world” topology, which is characterized by large clustering coefficients and short average path length [30, 40]. However, few studies have examined the hemispheric asymmetries from the perspective of the cerebral anatomical network, and little is known about the asymmetry of the connection patterns of brain regions, which may reflect the functional integration and interaction between different regions.

In this study, we first constructed the anatomical network for each subject by deterministic diffusion tensor tractography (DTT) technique, and then we applied graph theory approaches to examine the topological properties of bilateral brain regions of the network. To investigate the hemispheric asymmetries of the brain, statistical analyses were performed to reveal the brain regions with significant differences between bilateral topological properties. Furthermore, local structural connections were also investigated to examine the local asymmetries of some specific white matter tracts. From the perspective of both the global and local connection patterns, we identified the brain regions with hemispheric asymmetries.

## 2. Materials and Methods

### 2.1. Subjects

This study included 72 healthy adult subjects (42 males; mean age 23.4 ± 3.7 years; mean years of education 13.5 ± 4.7 years). All participants were right-handed according to the Edinburgh handedness inventory [41]. Each participant provided a written informed consent before MRI examinations and this study was approved by the Medical Research Ethics Committee of Xuanwu Hospital of Capital Medical University.

### 2.2. Data Acquisition

DTI was performed with a 3T Siemens Trio MR system using a standard head coil. Head motion was minimized with restraining foam pads provided by the manufacturer. Diffusion-weighted images were acquired employing a single-shot echo planar imaging (EPI) sequence in alignment with the anterior-posterior commissural plane. Integral Parallel Acquisition Technique (iPAT) was used with an acceleration factor of 2. Acquisition time and image distortion from susceptibility artifacts can be reduced by the iPAT method. The diffusion sensitizing gradients were applied along 12 nonlinear directions (*b* = 1000 s/mm^2^), together with an acquisition without diffusion weighting (*b* = 0 s/mm^2^). The imaging parameters were 45 continuous axial slices with a slice thickness of 3 mm and no gap, field of view = 256 mm × 256 mm, repetition time/echo time = 6000/87 ms, and acquisition matrix = 128 × 128. The reconstruction matrix was 256 × 256, resulting in an in-plane resolution of 1 mm × 1 mm. For each participant, a sagittal T1-weighted 3D image was also collected using a magnetization prepared rapid gradient echo (MP-RAGE) sequence. The imaging parameters for this were a field of view of 22 cm, repetition time/echo time = 24/6 ms, flip angle = 35°, and voxel dimensions of 1 mm × 1 mm × 1 mm.

### 2.3. Data Preprocessing

Eddy current distortions and motion artifacts in the DTI dataset were corrected by applying affine alignment of each diffusion-weighted image to the *b* = 0 image, using FMRIB's Diffusion Toolbox (FSL, version 3.3; http://www.fmrib.ox.ac.uk/fsl). After this process, the diffusion tensor elements were estimated by solving the Stejskal and Tanner equation [42, 43], and then the reconstructed tensor matrix was diagonalized to obtain three eigenvalues (*λ*_1_, *λ*_2_, and *λ*_3_) and eigenvectors. The fractional anisotropy (FA) of each voxel was calculated according to the following formula:

(1) $$\begin{array}{c} FA=\frac{\sqrt{{\left(\lambda_{1}-\lambda_{2}\right)}^{2}+{\left(\lambda_{1}-\lambda_{3}\right)}^{2}+{\left(\lambda_{2}-\lambda_{3}\right)}^{2}}}{\sqrt{2\left({\lambda_{1}}^{2}+{\lambda_{2}}^{2}+{\lambda_{3}}^{2}\right)}}. \end{array}$$

DTT was implemented with DTIstudio, Version 2.40, software (http://www.mristudio.org), by using the “fiber assignment by continuous tracking” method [44]. All tracts in the dataset were computed by seeding each voxel with FA greater than 0.2. Tractography was terminated if it turned an angle greater than 50 degrees or reached a voxel with FA less than 0.2 [45].

### 2.4. Construction of Anatomical Network

We constructed the anatomical network for each subject based on the fiber connectivity from deterministic DTT. The main procedures are as follows: First, the brain was automatically segmented into 90 cortical and subcortical regions (45 for each hemisphere; see Table 1) through AAL template (for details, see [32]). Briefly, the individual T1-weighted images were coregistered to the b0 images in the DTI space. The transformed T1 images were then nonlinearly transformed to the ICBM152 T1 template in the Montreal Neurological Institute (MNI) space. Inverse transformations were used to warp the AAL atlas from the MNI space to the DTI native space. Using this procedure, we obtained 90 cortical and subcortical ROIs, each representing a node of the network. Second, all fibers in the brain were obtained by deterministic fiber tractography. And then two nodes *u* and *v* are connected with an edge if there exist at least three fibers with end points in regions *u* and *v*; the threshold of three fibers was chosen to ensure that the average size of the biggest connected component of the network keeps 90 across all subjects. The number of fibers between regions was only used to indicate the existence/absence of the edge. Therefore, the binarized anatomical network for each subject was constructed and represented by a symmetric 90 × 90 matrix.

### 2.5. Network Analysis

We investigated the topological properties of the anatomical network at regional (nodal) levels. Regional properties were described in terms of degree (*K*_*i*_), shortest path length (*L*_*i*_), and betweenness centrality (*B*_*i*_) of the node *i*. Here, we provide brief, formal definitions of each nodal property used in this study.

#### 2.5.1. Degree

The degree *K*_*i*_ of a node *i* is defined as the number of connections to that node. Highly connected nodes have large degree. The degree *K*_*p*_ of a graph is the average of the degrees of all nodes in the graph:

(2) $$\begin{array}{c} K_{p}=\frac{1}{N}\underset{i\in G}{\sum }K_{i}, \end{array}$$

which is a measure to evaluate the degree of sparsity of a network.

#### 2.5.2. Shortest Path Length

The mean shortest path length *L*_*i*_ of a node *i* is

(3) $$\begin{array}{c} L_{i}=\frac{1}{N-1}\underset{i\neq j\in G}{\sum }L_{i,j}, \end{array}$$

in which *L*_*i*,*j*_ is the smallest number of edges that must be traversed to make a connection between the node *i* and the node *j*. The characteristic path length of a network is the average of the shortest path length between the nodes:

(4) $$\begin{array}{c} L_{p}=\frac{1}{N}\underset{i\in G}{\sum }L_{i}. \end{array}$$

*L*_*p*_ quantifies the ability of parallel information propagation or global efficiency (in terms of 1/*L*_*p*_) of a network [47].

#### 2.5.3. Betweenness Centrality

Betweenness centrality is widely used to identify the most central nodes in a network, which are associated with those nodes that act as bridges between the other nodes. The betweenness *B*_*i*_ of a node *i* is defined as the number of shortest paths between pairs of other nodes that pass through the node *i* [48, 49]. The normalized betweenness *b*_*i*_ was then calculated as

(5) $$\begin{array}{c} b_{i}=\frac{B_{i}}{\left(N-1\right)\left(N-2\right)}. \end{array}$$

The nodes with the largest normalized betweenness values were considered as pivotal nodes (i.e., hubs) in the network.

### 2.6. Reconstruction of White Matter Tracts

To further investigate the local asymmetries of the structural connections, we then reconstructed several major white matter tracts connecting different brain regions. Based on the anatomical knowledge of fiber projections, several studies have suggested the tracking protocols for the major white matter tracts [21, 50, 51]. According to the published tracking protocols, we reconstructed bilateral cingulum bundles (CB), optic radiation (OR), inferior frontooccipital fasciculus (IFO), inferior longitudinal fasciculus (ILF), arcuate fasciculus (AF), and uncinate fasciculus (UF) for each subject. Based on the reconstructed tracts for each subject, the mean FA of each fiber tract were calculated by averaging the FA values across the voxels that form the three-dimensional tracts derived from tractography.

### 2.7. Asymmetry Analysis

To analyze hemispheric differences in topological properties for brain regions, we computed the laterality ratio LI = (*L* − *R*)/(*L* + *R*) for each property (*K*_*i*_, *L*_*i*_, and *b*_*i*_). We tested the nullity of this ratio over the group using a nonparametric one-tailed sign test (*p* < 0.05 after Bonferroni correction for multiple comparisons; i.e., 90/2 = 45 pairs of regions). To analyze the hemispheric differences in structural properties for white matter tracts, we compared mean FA values and fiber numbers of each tract between left and right hemispheres by paired *t*-tests. For each tract, significant asymmetry of FA or fiber number was defined if *p* < 0.05. All the statistical analyses were performed with Matlab.

## 3. Results

### 3.1. Brain Regions with Hemispheric Asymmetries in Node Properties

**Figure 1:**
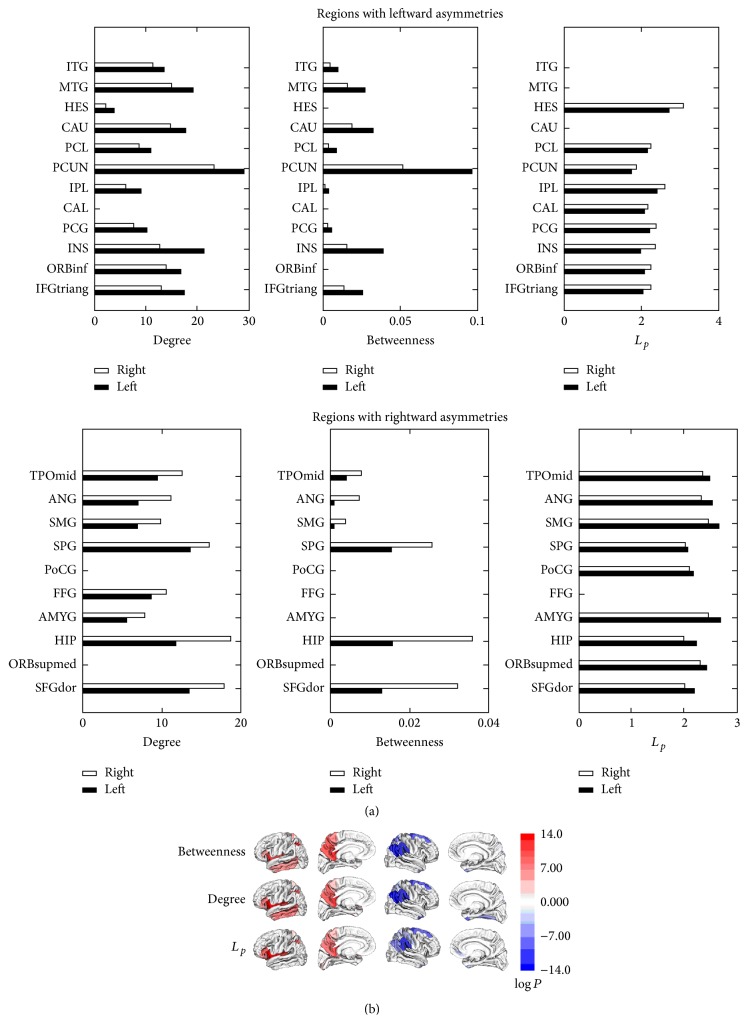
Cortical regions with hemispheric asymmetry in node properties. (a) Bars represent the mean values of the nodal property of brain regions with significantly hemispheric asymmetry (*p* < 0.05, corrected) (black: left; white: right). (b) 3D representation of the asymmetric cortical regions overlaid on the cortical surface (red: left > right for betweenness, degree, and 1/*L*_*p*_; blue: right > left for betweenness, degree, and 1/*L*_*p*_).

Based on the binary anatomical network constructed for each subject, we calculated the topological properties (*K*_*i*_, *L*_*i*_, and *b*_*i*_) of each node for each subject. Through statistical analyses for all subjects, we revealed some regions with hemispheric asymmetries in nodal properties (Figure 1). We defined leftward asymmetries with better topological properties in the left hemisphere than in the right, such as larger *K*_*i*_ and *b*_*i*_ and smaller *L*_*i*_ in the left. Similarly, rightward asymmetries were defined as regions with larger *K*_*i*_ and *b*_*i*_ and smaller *L*_*i*_ in the right hemisphere. From the results, we revealed some regions with hemispheric asymmetries in all three topological properties, such as leftward asymmetries in the inferior frontotriangular gyrus, insula, inferior parietal gyrus, and posterior medial cortex (paracentral lobule, precuneus, and posterior cingulate gyrus) and rightward asymmetries in the superior frontal gyrus, hippocampus, superior parietal gyrus, supramarginal gyrus, angular gyrus, and middle temporal pole (*p* < 0.05 after Bonferroni correction).

### 3.2. Structural Asymmetries of the White Matter Tracts

**Figure 2:**
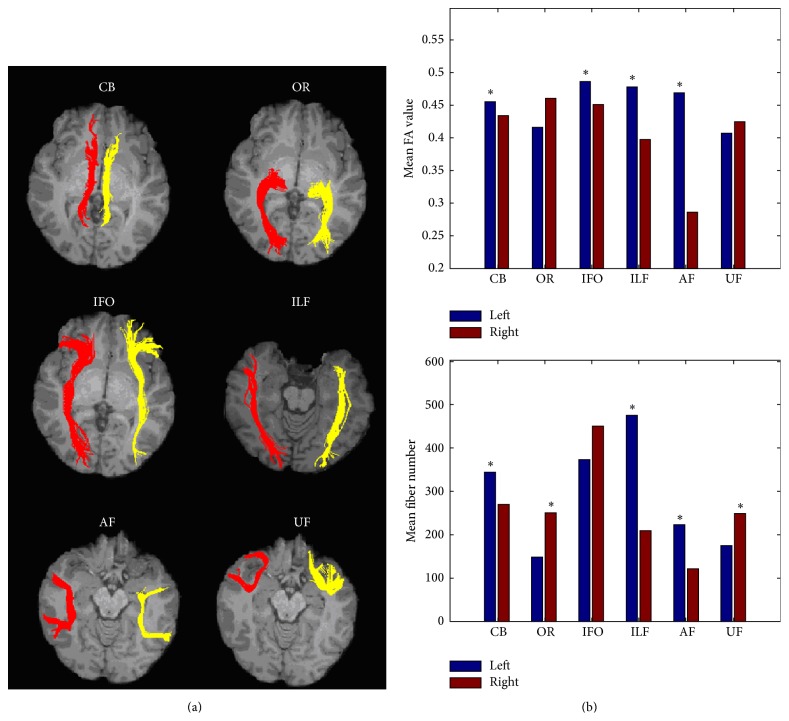
Structural asymmetries of major white matter tracts. (a) Reconstructed bilateral white matter tracts: cingulum bundles (CB), optic radiation (OR), inferior frontooccipital fasciculus (IFO), inferior longitudinal fasciculus (ILF), arcuate fasciculus (AF), and uncinate fasciculus (UF) (red: left; yellow: right). (b) Between-hemisphere differences for the structural properties of the white matter tracts (*∗*: significant differences at *p* < 0.05).

For each subject, we can successfully reconstruct most of the bilateral white matter tracts (Figure 2(a)). However, the right AF is difficult to be tracked out for some subjects (15 out of 72). From Figure 2(b), we can see that several white matter tracts exhibit hemispheric asymmetries in both the microstructural (FA value) and macrostructural (fiber number) properties, such as CB, ILF, and AF (*p* < 0.05, uncorrected).

## 4. Discussion

In this study, we investigated the hemispheric asymmetries of the human brain from the perspective of the cerebral anatomical network constructed from DTI data. By comparing bilateral topological properties, we revealed some brain regions with significant leftward or rightward asymmetries, which indicated the asymmetric connection patterns of these regions. Moreover, the structural properties of some local white matter tracts also exhibit hemispheric asymmetries, which indicated the asymmetries of local connections. It suggested that the structural organizations of the human brain are asymmetric from both the global and local connection patterns. Then, the functional meanings of these structural asymmetries should be discussed.

### 4.1. Hemispheric Asymmetries in Node Properties of the Anatomical Network

Previous studies of hemispheric asymmetries in the human brain focused on the structures or functions of some local regions [1, 2, 20, 52, 53]. In this study, we explored the hemispheric asymmetries from the connection patterns between different brain regions, by comparing the topological properties of nodes between bilateral hemispheres in the anatomical network.

Different nodal properties reflect different aspects of the node in the network. In this study, we chose three topological properties, degree, normalized betweenness centrality, and shortest path length, to analyze the hemispheric asymmetries of the anatomical network. Degree means the number of direct connections to the node. Larger degree means more structural connections to other brain regions in the binary anatomical network. Betweenness centrality reflects the importance of the node, and a node with high centrality is thus crucial to efficient communication [48, 49]. Shortest path length quantifies parallel information propagation or global efficiency (in terms of 1/*L*_*i*_) of the node, and smaller *L*_*i*_ means higher global efficiency of the parallel information transfer [47]. Although these three properties interrelated with each other, they reflect different aspects of the node in the network. Therefore, a node with a larger degree, a higher centrality, and a smaller shortest path length will play a more important role in the network. Then we suggested that the asymmetric properties of the regions in bilateral hemispheres indicate the lateralization of these regions in the anatomical network.

Since the regions with hemispheric asymmetries in nodal properties were revealed, we categorized these regions by their functions as follows.

*Regions with Leftward Asymmetries*

1. Language and auditory function: middle and inferior temporal gyrus [54, 55], caudate nucleus [56, 57], Heschl's gyrus [58, 59], and triangular and orbital part of inferior frontal gyrus [60, 61].
2. Visual function: middle and inferior temporal gyrus [62, 63], calcarine fissure, and surrounding cortex [62, 64].
3. Emotion, sensation, and addiction: insula [65, 66].
4. Association cortex: paracentral lobule, precuneus, posterior cingulate gyrus, and inferior parietal gyrus.

*Regions with Rightward Asymmetries*

1. Spatial attention: angular and supramarginal gyrus [67–69].
2. Face recognition: fusiform gyrus [70, 71].
3. Emotion and memory: hippocampus and amygdala [68, 72–74].
4. Association cortex: superior and middle frontal gyrus, superior parietal gyrus, and middle temporal pole.

From the results, we can see that regions with leftward asymmetries are mainly related to language, visual processing, and sensory functions. Regions with rightward asymmetries are mainly related to the functions of spatial attention, face recognition, emotion, and memory. Some regions in the association cortex with multiple functions also exhibit leftward or rightward asymmetries in the nodal properties.

Combined with the findings of some previous studies, we speculated that the topological asymmetries in the anatomical network are likely to form the structural substrate of different functional principles of information processing in the two hemispheres. Since Paul Broca's discovery in 1861, the notion of left hemisphere specialization for language has been established [11]. Recently, more and more studies revealed the leftward asymmetries in the size of regions involved in language and auditory processing, such as planum temporale [4–7], sylvian fissure [8], and Heschl's gyrus [4, 9, 10], which may supply the anatomic basis of the functional lateralization in the human brain. In this study, the most obvious finding is the leftward asymmetries in several language related regions, such as triangular and orbital areas of inferior frontal gyrus, middle and inferior temporal gyrus, and Heschl's gyrus. The results dovetailed with the prevailing notion that the left hemisphere is the dominant hemisphere for language [75, 76]. It may also suggest that not only the structural asymmetries of these regions, but also the asymmetric connection patterns of these regions support the functional lateralization of the human brain.

Another finding is the rightward asymmetries of angular and supramarginal gyrus, which located in the temporoparietal junction. The right angular and supramarginal gyrus have been widely implicated in the functions of spatial attention [67–69]. Previous functional MRI studies have also revealed that right temporoparietal junction plays a dominant role in actual implementation of spatial attention by functional connectivity analysis [77, 78]. Therefore, this result corresponded with the right hemispheric specializations for visuospatial functions [75, 79–81].

Some subcortical structures in the limbic system, such as hippocampus and amygdala, were also revealed with rightward asymmetries in the topological properties. Hippocampus plays an important role in memory and spatial navigation [68, 74], and amygdala performs a primary role in the processing of memory and emotion [72, 73]. Then, in this study, the rightward asymmetries in topological properties of hippocampus and amygdala may suggest that the right hemisphere is more prominent in the functions of emotion and memory. It is also consistent with the findings of some structural MRI studies which indicated that the hippocampus and amygdala are rightward asymmetric based on the volume measurements [82].

Besides the above results, we revealed some regions with hemispheric asymmetries in all three topological properties, such as leftward asymmetries in three regions of posterior medial cortex (paracentral lobule, precuneus, and posterior cingulate gyrus), inferior parietal gyrus, and insula and rightward asymmetries in middle temporal pole, superior parietal gyrus, and superior frontal gyrus. Most of these regions are located in the association cortex, which plays a central role in receiving convergent inputs from multiple cortical regions [68]. To be mentioned, three continuous regions in the posterior medial cortex have been revealed as the structure core of the cerebral cortex by a diffusion MRI study [36]. However, no study has examined the functional or structural asymmetries of these core regions yet. Then, in this study, it is the first time to reveal the leftward asymmetries of the core regions in the anatomical network.

Of note, abnormal asymmetric patterns of brain structure or function have been implicated in some psychiatric disorders, such as schizophrenia, and the extent of altered asymmetry is related to the symptoms of the patients [83]. Therefore, we speculated that the asymmetric topology of brain networks would also change under various conditions with mental diseases and may supply as sensitive biomarkers for early disease detection, which should be further investigated.

### 4.2. Structural Asymmetries of the White Matter Tracts

Based on the tractography results of six major white matter tracts, we analyzed the structural asymmetries of these tracts in mean FA values and fiber numbers. Previous DTI studies have identified the anatomical asymmetries of some fiber tracts, such as leftward asymmetries of the arcuate fasciculus [2, 3, 19–21]. As one of the most important language pathways, arcuate fasciculus starts from Broca's area in inferior frontal gyrus and projects into the middle and inferior temporal gyrus [84]. In this study, we revealed the leftward asymmetries of AF from both the micro- and macrostructural properties. The leftward asymmetry of AF corresponds with the leftward asymmetries of the triangular area of inferior frontal gyrus, middle and inferior temporal gyrus revealed by the topological analysis of the anatomical network. These results suggest that the language related regions exhibit leftward asymmetries from both the global and local anatomical connection patterns. We speculated that these structural asymmetries may provide the anatomical substrate of language related functional lateralization of the human brain. Besides the leftward asymmetries of AF, the CB and ILF are also leftward asymmetric in both mean FA values and fiber numbers. The cingulum bundles have been investigated in several previous DTI studies, and the leftward asymmetries in fiber integrity were identified by different methods [15, 16, 85]. The inferior longitudinal fasciculus, which connects the temporal lobe and occipital lobe, plays an important role in visual memory [86, 87] and is considered as an indirect pathway of language semantic processing [88]. This is the first time leftward asymmetry of ILF is revealed and it may provide new information for future studies. The inferior frontooccipital fasciculus, which connects the posterior occipital areas and the orbitofrontal region, is a direct pathway of language semantic processing [88]. A previous DTI study has reported the leftward asymmetries in the fiber integrity of IFO and has suggested that the structural asymmetries of the tract correspond with the hemispheric dominance for language [21]. Additionally, we found that the optic radiation and uncinate fasciculus are rightward asymmetric in fiber numbers. However, the functional meaning of these asymmetries should be examined in future studies.

As both the global (structural connectome) and local (FA) measures were investigated in the present study, some results can be cross-validated by different measures. For example, we found that both the language related regions and WM tracts exhibited significantly leftward asymmetries. However, global and local measures may represent different physiological meanings. The local measure, such as FA, reflects the white matter integrity or the consistency of fiber orientation at microstructural level, while the nodal properties of brain connectome, such as nodal efficiency, are an integrated metric of global information flow capacity and related to all of the nodal connections, which consist of a specific tract or several tracts together. Therefore, the findings from network analysis can supply more comprehensive information than the traditional regional and local investigations from a system level.

### 4.3. Methodological Issues

The most essential elements of a network are the nodes and edges. The definition of the nodes and edges has a great effect on the constructed network and the analysis results. Therefore, we need to address some methodological issues about how we carried out the network construction.

First, we applied the AAL template to define the nodes for each subject's network. The AAL template was taken from a MNI single-subject brain [46]. The biggest limitation of this template is the absence of anatomical lateralization of some regions, such as leftward asymmetry of the planum temporale for the vast majority of right-handers [46]. This limitation will affect the results of the topological asymmetries of the anatomical network in this study. In future studies, a more fine parcellation representation, which is defined at a voxel population level rather than a regional level to partition the cerebral cortex into thousands of regions [36], should be employed to investigate the asymmetries of the brain network, in order to localize the asymmetric topological organizations more accurately.

Second, we employed deterministic DTT to define the edges of the anatomical network. However, the “fiber crossing” problem is a limitation of deterministic tractography algorithms, because the tracking always stops when it reaches fiber crossing regions with low factional anisotropy values [89]. This will result in the loss of some existing fibers and hence some edges of the network. Another limitation of deterministic tractography, especially for long-distance fiber bundles, is erroneous tracking results due to noise and resolution limitations [89]. To solve this issue, several researchers have used probabilistic fiber tracking algorithms [90–92]. By modeling a probability distribution of the fiber orientations within a voxel, these statistical methods can identify fiber connections missed by deterministic tracking approaches. However, the number of gradient directions in our diffusion dataset is not sufficient to accurately estimate the probability density function of the fiber orientations. Therefore, future studies with more advanced diffusion imaging techniques or tractography methods could yield a more complete and accurate anatomical network for each subject.

Another issue about the choice of a binary or weighted network needs addressing. For a weighted network, a challenge is to decide on the most representative measure of structural connectivity. Several candidate measures, such as fiber numbers, mean fiber length, fiber density, and mean fraction anisotropy, can be selected as the connectivity measure [25, 38, 39, 93]. But the physiological meaning of these measures is unclear. It is also hard to validate which measure describes the information transfer of neural signals most accurately. In this work, we constructed the binary network by just taking into consideration the existence/absence of regional connections. However, a weighted network with a proper connectivity measure may better reflect the topological asymmetries of the network.

Besides the above methodology limitations, some other important issues should be investigated in the future. First, as the sex effects on the topological organization of brain networks have been suggested [94], the sex differences in the network asymmetry should be examined in the future. Second, due to the relatively small sample size in the present study, other independent datasets with high quality MRI acquisition and larger samples, such as Human Connectome Project (HCP) datasets [95], should be employed to validate the current results.

## 5. Conclusion

In this study, we have analyzed the hemispheric asymmetries from the perspective of the whole-brain anatomical network and revealed the topological asymmetries of some brain regions, which indicated the asymmetric connection patterns of these regions at the global level. Moreover, we found the structural asymmetries of some local anatomical connections between regions, and the structural asymmetries of the white matter tracts are interrelated with the topological asymmetries of the brain regions. We speculated that the asymmetric connection patterns of brain regions might reflect the functional lateralization of the human brain.

## Figures and Tables

**Table 1:** Cortical and subcortical regions of interest defined in the study.

Index	Regions	Abbr.
(1, 2)	Precentral gyrus	PreCG
(3, 4)	Superior frontal gyrus, dorsolateral	SFGdor
(5, 6)	Superior frontal gyrus, orbital part	ORBsup
(7, 8)	Middle frontal gyrus	MFG
(9, 10)	Middle frontal gyrus, orbital part	ORBmid
(11, 12)	Inferior frontal gyrus, opercular part	IFGoperc
(13, 14)	Inferior frontal gyrus, triangular part	IFGtriang
(15, 16)	Inferior frontal gyrus, orbital part	ORBinf
(17, 18)	Rolandic operculum	ROL
(19, 20)	Supplementary motor area	SMA
(21, 22)	Olfactory cortex	OLF
(23, 24)	Superior frontal gyrus, medial	SFGmed
(25, 26)	Superior frontal gyrus, medial orbital	ORBsupmed
(27, 28)	Gyrus rectus	REC
(29, 30)	Insula	INS
(31, 32)	Anterior cingulate and paracingulate gyri	ACG
(33, 34)	Median cingulate and paracingulate gyri	DCG
(35, 36)	Posterior cingulate gyrus	PCG
(37, 38)	Hippocampus	HIP
(39, 40)	Parahippocampal gyrus	PHG
(41, 42)	Amygdala	AMYG
(43, 44)	Calcarine fissure and surrounding cortex	CAL
(45, 46)	Cuneus	CUN
(47, 48)	Lingual gyrus	LING
(49, 50)	Superior occipital gyrus	SOG
(51, 52)	Middle occipital gyrus	MOG
(53, 54)	Inferior occipital gyrus	IOG
(55, 56)	Fusiform gyrus	FFG
(57, 58)	Postcentral gyrus	PoCG
(59, 60)	Superior parietal gyrus	SPG
(61, 62)	Inferior parietal but supramarginal and angular gyri	IPL
(63, 64)	Supramarginal gyrus	SMG
(65, 66)	Angular gyrus	ANG
(67, 68)	Precuneus	PCUN
(69, 70)	Paracentral lobule	PCL
(71, 72)	Caudate nucleus	CAU
(73, 74)	Lenticular nucleus, putamen	PUT
(75, 76)	Lenticular nucleus, pallidum	PAL
(77, 78)	Thalamus	THA
(79, 80)	Heschl gyrus	HES
(81, 82)	Superior temporal gyrus	STG
(83, 84)	Temporal pole: superior temporal gyrus	TPOsup
(85, 86)	Middle temporal gyrus	MTG
(87, 88)	Temporal pole: middle temporal gyrus	TPOmid
(89, 90)	Inferior temporal gyrus	ITG

Note: the regions are listed in terms of a prior template of an AAL atlas [46].
